# Stability of emergency medications during extreme cold: a controlled environmental study

**DOI:** 10.1186/s13049-025-01509-w

**Published:** 2025-12-05

**Authors:** David Sohm, Johannes Moeckel, Volker Wenzel, Verena Angerer, Giulia Roveri, Simon Rauch, Roland Albrecht, Urs Pietsch

**Affiliations:** 1 Division of Anaesthesiology, Rescue- and Pain Medicine, HOCH Health Ostschweiz, Cantonal Hospital St. Gallen, St. Gallen, Switzerland; 2 Division of Perioperative Intensive Care Medicine, HOCH, Cantonal Hospital St. Gallen, St. Gallen, Switzerland; 3 Swiss Air-Ambulance, Rega (Rettungsflugwacht / Guarde Aérienne), Zurich, Switzerland; 4 Department of Emergency Medicine, Inselspital, University Hospital Bern, Bern, Switzerland; 5 Institute of Forensic Medicine, Forensic Toxicology, Cantonal Hospital St. Gallen, St. Gallen, Switzerland; 6 Institute of Mountain Emergency Medicine, Eurac Research, Bolzano, Italy; 7 Department of Anesthesiology and Intensive Care Medicine, Merano Hospital, Merano, Italy; 8 Department of Anaesthesiology and Intensive Care Medicine, Friedrichshafen Regional Hospital, Friedrichshafen, Germany; 9 Department of Anesthesiology, University of Florida, Gainesville, FL USA

**Keywords:** HEMS, Emergency medications, Drug stability, Alpine rescue, Mountain medicine, Prehospital care, Drug storage

## Abstract

**Background:**

Conditions of extreme cold, encountered during mountain and glacial rescue operations, pose challenges for the storage of emergency medications. Understanding how repeated exposure to extreme cold and ambient temperatures affects drug stability is essential for safe prehospital care.

**Methods:**

A controlled environmental study was conducted at the terraXcube, a high-fidelity climate simulation facility at Eurac Research in Bolzano, Italy. The study drugs included Acetazolamide, Amiodarone, Dexamethasone, Epinephrine, Ketamine, Naloxone, Norepinephrine and Rocuronium. Drug ampoules were stored within an insulated storage bag, placed inside a regular mountain rescue backpack. This backpack was then used in a high-fidelity training scenario under conditions of extreme cold. The ampoules remained sealed throughout the experiment. The drugs underwent six cycles of exposure, consisting of 45 min at -15 °C followed by 15 min at + 18 °C, simulating temperature fluctuations during repeated alpine rescue operations. Stability was assessed through visual inspection for physical changes (e.g., crystallization, phase separation) and chemical analysis using mass spectrometry, with results expressed as a percentage of the reference concentration.

**Results:**

Visual inspections revealed no overt physical alterations. Mean ± standard deviation (SD) of remaining concentrations ranged from 92.1 ± 1.3% (acetazolamide) to 101.8 ± 7.1% (dexamethasone), with all eight medications retaining ≥ 90% of their labeled concentrations.

**Conclusion:**

Emergency medications can remain chemically stable under extreme cold conditions when stored in sealed, insulated packaging. While our study simulated prehospital conditions without direct environmental exposure, these findings support the feasibility of extended storage and transport of emergency medications in challenging field settings. Further research should assess the impact of direct environmental exposure and evaluate additional stability parameters to optimize storage protocols in real-world scenarios.

**Trial registration:**

Not applicable.

**Supplementary Information:**

The online version contains supplementary material available at 10.1186/s13049-025-01509-w.

## Background

Ensuring the stability of emergency medications during extreme environmental conditions is critical for patient safety in prehospital settings such as helicopter emergency medical services (HEMS) and alpine rescue, where temperatures may fall far below recommended storage ranges. Although previous studies have shown that certain medications maintain potency across a range of temperatures, the impact of sustained subzero conditions and repeated exposure to very cold temperatures remains insufficiently understood.

Drug degradation is especially concerning for temperature-sensitive formulations prone to crystallization or phase separation in subzero conditions, and limited access to replacement medications in remote, high-altitude areas further underscores the need to preserve drug potency. Prior investigations have mostly focused on sealed storage within emergency medical services (EMS) vehicles - as seen in studies by Welter et al. [1] and Pietsch et al. [2] - and did not simulate true ambient extremes under sustained subzero exposure that mimics alpine rescue conditions.

Official guidance by the UIAA Medical Commission (MedCom Recommendation No. 10: *Drugs at Altitude*) already emphasizes that both heat and cold can significantly alter the physical and chemical stability of drugs, with reported in-bag temperatures ranging from − 40 °C to + 80 °C. It warns that frozen ampoules may develop hairline cracks or undergo irreversible degradation, particularly in protein-containing and emulsion-based formulations, and that previously frozen solutions should ideally be replaced [3]. However, these recommendations are largely based on expert consensus and limited empirical data, highlighting a persistent evidence gap regarding quantitative stability under controlled and realistic alpine conditions.

More recent literature continues to cite the UIAA recommendations as the main source of guidance but notes that systematic experimental validation of these assumptions, especially under repeated subzero exposure in insulated rescue packs, remains insufficient. Addressing this gap is essential to refine evidence-based storage protocols for emergency drugs used in mountain rescue and other extreme environments.

A remaining drug concentration of at least 90% label claim has been determined as the acceptable limit of retained drug stability by the European Medicines Agency and the United States Pharmacopeia. Therefore, our goal was to determine whether emergency medications retain ≥ 90% of their labeled concentration when exposed to conditions that challenge standard storage recommendations.

## Materials and methods

### Study design

In this controlled environmental study, we analyzed medications labeled for storage at room temperature or during refrigeration (Table 1) that were stored in insulated storage bags and exposed to six repeated alternating temperature cycles (-15 °C / 5° F for 45 min, + 18 °C / 64° F for 15 min). Physical integrity of the ampoules was inspected after each cycle, whereas the chemical stability was analyzed after completion of the final (sixth) cycle. The duration of cold exposure phases was chosen to approximate the typical length of an alpine rescue operation.

**Table 1:**
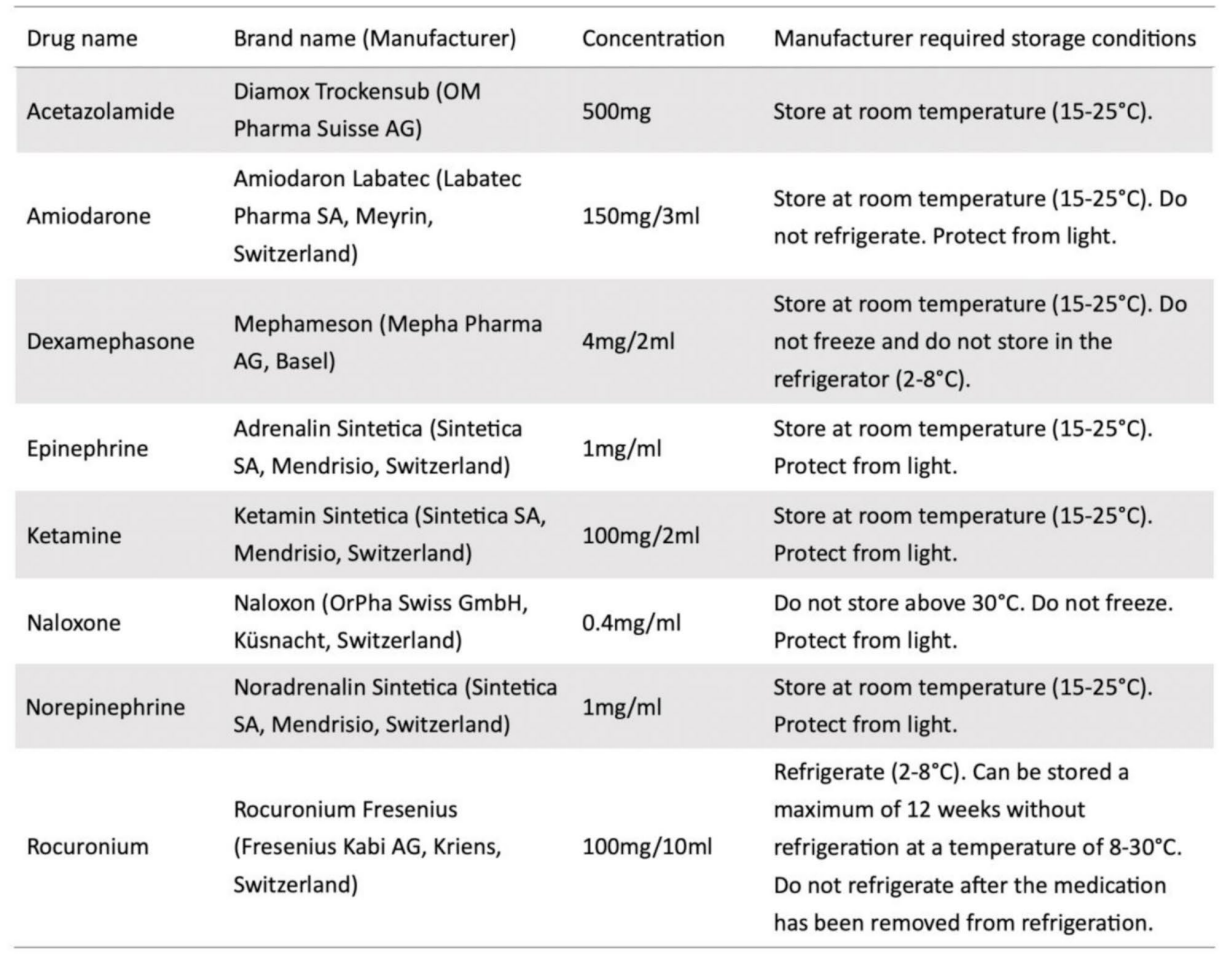
Study drugs. Manufacturer information and recommended storage conditions

### Setting and drug storage

**Fig. 1:**
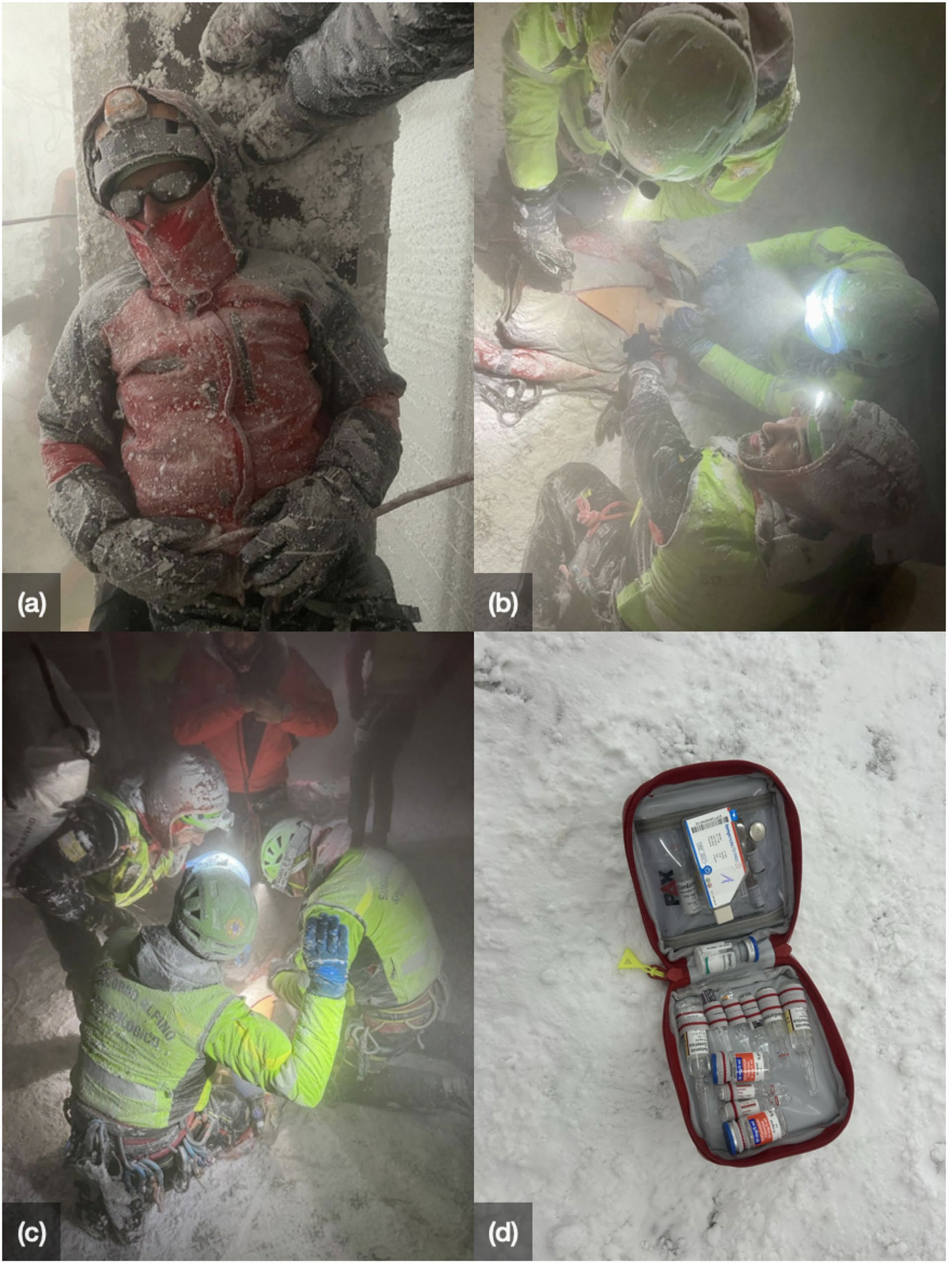
Simulation of prehospital emergency care under high-fidelity scenarios in the terraXcube. (**a**) Patient simulating a casualty during mountain rescue. (**b**) Rescue team performing advanced life support on a mannequin at -15 °C and 10 m/s wind inside the chamber. (**c**) Team operating in full gear under simulated alpine conditions. (**d**) PAX^®^ insulated storage bag with study medication and the temperature data logger tempmate^®^ S1 Pro. Image source (**a-d**): Author photos, terraXcube, Italy, 2024

The study was conducted at the terraXcube, a high-fidelity climate simulation facility operated by Eurac Research in Bolzano, Italy (Fig. 1). This facility allows for precise, reproducible control of extreme environmental conditions - including temperature, humidity, and wind - enabling realistic simulations of harsh, real-world scenarios [4].

Testing coincided with a mountain rescue training simulation inside the terraXcube. Six mountain rescue teams each performed a rescue scenario at -15 °C, with wind speeds of 10 m/s (36 km/h) and relative humidity between 5 and 15%, lasting 45 min per session.

All medications were stored in ampoules within an insulated storage bag (PAX Pro Series-ampoule kit narcotic substances 9, Model no. 224370301, X-CEN-TEK GmbH, Wardenburg, Germany), placed inside a standard mountain rescue backpack. During the rescue simulations, the ampoule storage bag mostly remained closed and inside the backpack. It was only opened as needed. Between sessions, the backpacks were exposed to + 18 °C for 15 min.

A temperature data logger (tempmate S1 Pro - No longer available, tempmate GmbH, Heilbronn, Deutschland) (Fig. 1) placed inside the storage bag recorded internal temperatures every 10 min throughout the simulation period.

As shown in Fig. 1, the insulated storage bag was occasionally opened to simulate real-world medication retrieval during missions. However, it remained closed as much as possible and was not left open in subzero environments - reflecting standard alpine rescue practice.

This setup closely mirrored real-world prehospital storage conditions, ensuring the medications were not directly exposed to the chamber environment but were instead subjected to temperature fluctuations through typical field-use storage, as encountered in actual mountain rescue operations. The selected medications represent some of the most relevant drugs used in prehospital emergency care, including advanced cardiac life support, analgesia, and high-altitude medicine.

### Testing

Two ampoules of each medication, along with fresh reference ampoules (stored per manufacturer’s instructions in the hospital pharmacy) were inspected visually before and after each cycle for crystallization, phase separation, vial breakage, or discoloration. Any macroscopic alterations not described in the manufacturer’s specifications would have led to sample exclusion. These inspections were first performed onsite by team members immediately each time after the ampoules were removed from the cold chamber and repeated by the analytical lab team prior to chemical processing. Ampoules were manually handled and examined under ambient indoor lighting conditions outside the cold chamber. All ampoules remained sealed and intact throughout the experiment, no intermediate samples were taken.

We did not directly measure whether the medications themselves froze during cold exposure. No visible freezing or formation of ice crystals was observed. However, given the subzero temperatures and internal bag recordings freezing cannot be definitively ruled out.

Chemical stability was assessed by high-performance liquid chromatography–diode array–tandem mass spectrometry (HPLC-DAD-MS/MS).

Each sample was analyzed in three replicates, and measured concentrations were expressed as a percentage of the corresponding reference ampoule (set to 100% label claim). A remaining concentration ≥ 90% - defined a priori - was used as the criterion for chemical stability.

Detailed analytical parameters are available as a supplementary file.

### Statistical analysis

For each ampoule, the arithmetic mean of the three HPLC-DAD-MS/MS replicates was calculated to yield a single concentration estimate per drug per cycle. Each estimate was then divided by the mean concentration of its paired reference ampoule and multiplied by 100 to express stability as a percentage of label claim.

Normality of the percentage data was assessed by visual inspection of quantile–quantile plots. All analyses were performed in R (version 4.1.1; R Foundation for Statistical Computing). Results are reported as mean and range for each medication. *P* < .05 was considered to be statistically significant.

## Results

A total of 16 exposed ampoules were tested (2 per drug), with each analyzed in triplicate. Results were compared against 16 matched reference ampoules.

Visual inspections of all medication samples revealed no signs of freezing, breakage, phase separation, or other macroscopic alterations after exposure to extreme environmental conditions. Internal storage bag temperatures fluctuated within a span of approximately 32 °C over the course of the experiment.

**Fig. 2:**
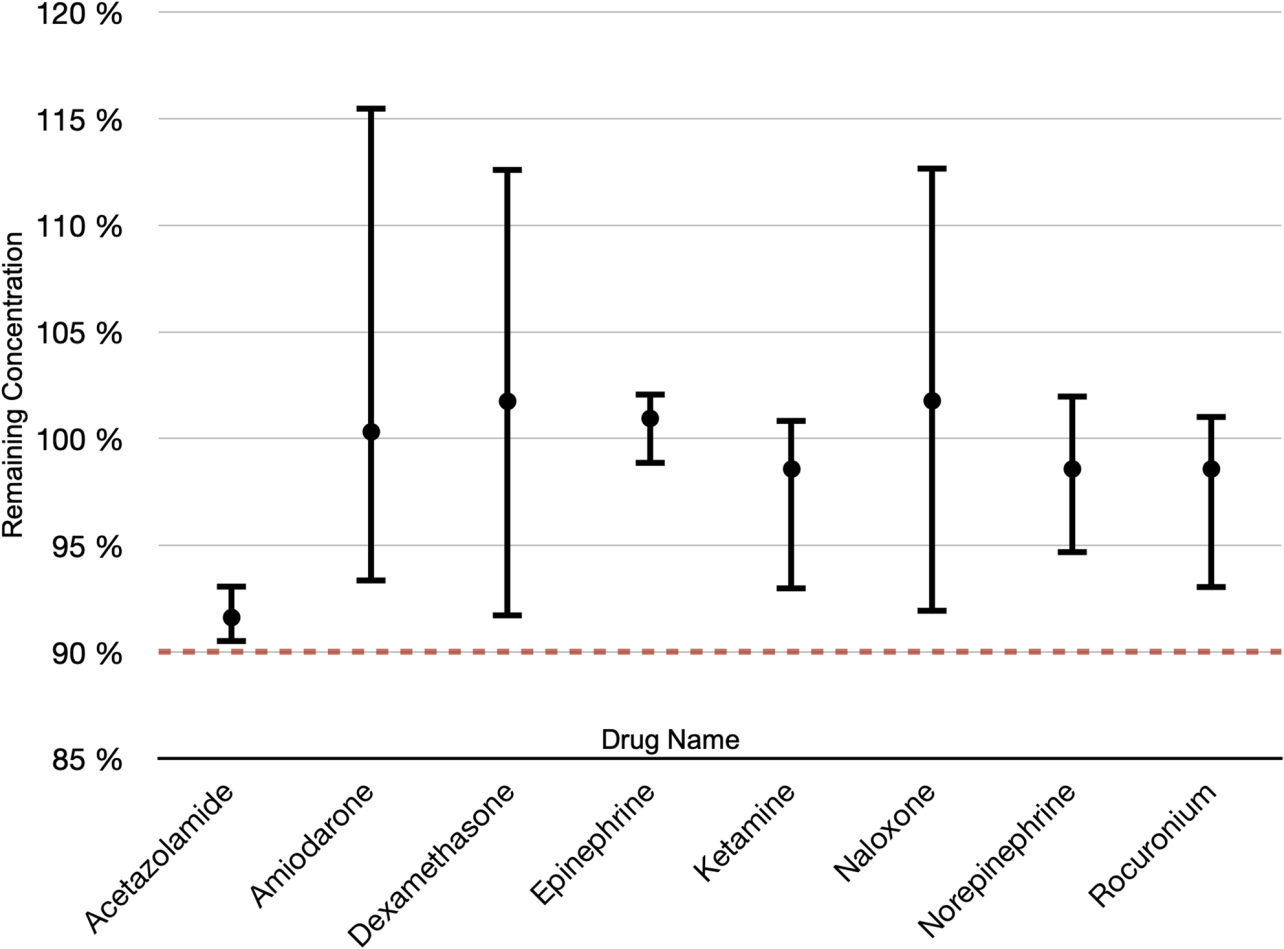
Study drug performance. Remaining concentration of active pharmaceutical ingredient after repeated exposure to -15 °C for 45 min, interspersed with 15 min at + 18 °C. Bars show min-max values with means indicated; the red line marks the 90% stability threshold

Chemical analysis demonstrated mean remaining concentrations of acetazolamide 92.1% (90.7–93.2%), amiodarone 100.5% (93.2–115.7%), dexamethasone 101.8% (91.9–112.8%), epinephrine 101.0% (99.0–102.3%), ketamine 98.9% (93.1–100.9%), naloxone 101.8% (91.9–112.8%), norepinephrine 99.0% (94.8–102.1%), and rocuronium 99.0% (93.0–101.0%).  All drugs thus remained within the predefined 90% stability threshold, confirming preserved chemical integrity during repeated exposure to extreme cold (Fig. 2).

## Discussion

This trial demonstrated that eight commonly used emergency medications maintained their chemical stability when subjected to repeated cycles of subzero and ambient temperatures within insulated rescue storage bags. Under realistically simulated alpine conditions, all drugs retained more than 90% of their labeled concentrations, and no visible, physical changes were detected. These findings provide empirical support that the short-term storage and transport of sealed ampoules in insulated packaging can preserve drug integrity even during extended missions in cold, high-altitude, or glacial environments.

While previous research has explored temperature effects on medications stored in EMS vehicles or helicopters [1, 2], few studies have examined drug stability under subzero conditions, representative of alpine rescue operations. By reproducing the alternating cold-warm cycles encountered during real rescue deployments, our study provides quantitative data extending the scope of knowledge to field conditions outside the protection of a vehicle. These results help to close a key evidence gap as the UIAA Medical Commission acknowledges that recommendations and warnings concerning damage to ampoules or drug degradation in extreme cold are largely based on expert consensus rather than controlled experiments [3]. 

By focusing on the timeframe of a typical alpine rescue mission our findings are particularly applicable to air-, and ground-based emergency medical services in alpine terrain. However, drugs carried by medical practitioners in the field of expedition medicine are sometimes faced with week-long exposure to extreme temperatures, warranting further research and exploration in this field.

Our results suggest that, for the timeframe and drugs tested, current storage practices using insulated pouches within rescue backpacks are sufficient to maintain chemical stability despite repeated exposure to subzero ambient temperatures. This evidence supports the continued use of such methods and may reduce unnecessary concern or premature disposal of ampoules exposed to transient cold.

However, not all medications can be expected to behave similarly. Protein-based or emulsion formulations such as Insulin, Propofol, and Etomidate are known to be more vulnerable to cold-induced degradation, phase separation, or droplet formation, which may compromise both safety and efficacy. Previous microscopic studies have demonstrated that lipid emulsions can develop sub-visible droplets after cold exposure, rendering them unsuitable for intravenous administration [5]. These formulations were deliberately excluded from our study but merit focused investigation in future work to establish a more comprehensive guidance for field storage.

Several methodological limitations must be considered. The study was conducted in a controlled simulation environment, which, while realistic, cannot reproduce all physical stressors encountered in actual rescue missions. Factors such as vibration, ultraviolet light, or humidity were outside the scope of our experimental design. Freezing of the ampoule contents was not directly verified, and although internal bag temperatures dropped below zero, phase changes were not recorded in real time. Temperature logging occurred at ten-minute intervals and may have missed brief peaks or troughs during handling. In this trial, chemical and physical stability were assessed as surrogate parameters for drug stability; microbiological or pharmacodynamic changes were not examined. A complete assessment would have to include analysis for physical integrity such as microscopy or single optical particle sensing, as well as in vivo testing of absorption, distribution and therefore bioavailability at the effect site. Despite these constraints, the high reproducibility and tightly controlled environmental parameters provide robust initial evidence for the stability of these compounds under simulated field conditions.

Overall, the consistency of concentration values across all tested medications indicates that brief, repeated exposure to very cold temperatures does not compromise chemical integrity when ampoules remain sealed and insulated. This finding strengthens the empirical basis for existing alpine and prehospital storage recommendations and underscores the resilience of standard emergency drug formulations under environmental stress.

## Conclusion

Commonly used emergency medications, including agents for analgesia, airway management, and advanced life support, remain chemically stable during repeated exposure to extreme cold when stored in sealed ampoules within rescue bags. These results provide controlled experimental evidence supporting current alpine and prehospital storage practices as safe for short-term use. Future research should focus on temperature-sensitive formulations such as emulsions and protein-based drugs, incorporate real-time freezing verification, and assess longer-term storage stability to further refine evidence-based guidelines for medication handling in extreme environments.

## Supplementary Information

Below is the link to the electronic supplementary material.

Supplementary Material 1

## Data Availability

The data used to support the findings of this research are included within the article. Detailed analytical parameters are available as a supplementary file. Any additional data regarding the case presented are available from the corresponding author upon reasonable request.

## Abbreviations

EMS: Emergency Medical Services
HEMS: Helicopter Emergency Medical Services
HPLC–DAD–MS/MS: High–Performance Liquid Chromatography–Diode Array Detector–Tandem Mass Spectrometry
SD: Standard Deviation
