# Supplementary material for: Stability of emergency medications during extreme cold: a controlled environmental study
Source: Scand J Trauma Resusc Emerg Med. 2025 Dec 5;34:5. doi: 10.1186/s13049-025-01509-w (PMC12797534; doi:10.1186/s13049-025-01509-w)
Supplement: Supplementary file 1 — Supplementary Material 1 [file 13049_2025_1509_MOESM1_ESM.docx]

Stability of Emergency Medications During Extreme Cold: A Controlled Environmental Study

***Supplementary analytical data***

**Testing**

Each trial medication was represented by 2 exposed ampoules. A third ampoule was used as a reference. After the conclusion of six 45-minute training exercises at -15°C, followed by a 15-minute brake at +18°C in between each exercise, the trial ampoules were removed from the storage bag and transported, under room temperature conditions, to the laboratory. Drug ampoules used as reference samples were fresh ampoules, stored according to their specific recommended storage conditions at the hospital pharmacy, and were delivered directly to the testing laboratory. Before testing, the ampoules were checked visually for breakage or any changes in color or viscosity not described in the manufacturers’ instructions. Stability testing was performed using a quantitative confirmatory analysis utilizing high-performance liquid chromatography-diode array-tandem-mass spectrometry (HPLC-DAD-MS/MS, 20ADXR and SPD M20A). A total of three HPLC-MS runs were performed for each study ampoule. All measurements were recorded as the percentage of remaining drug concentration in relation to the reference samples. The reference samples were set as a drug concentration of 100% label claim. Study drugs were considered stable at a minimum remaining drug concentration of 90% of the label claim.

**Analysis:**

Quantitative analysis was performed using a high-performance liquid chromatography-diode array-tandem-mass spectrometry (HPLC-DAD-MS/MS, 20ADXR and SPD M20A, Shimadzu; 3200 QTrap, Sciex). Separation was performed using a Zorbax Eclipse XDB-C8 4.6 x 150 mm, 5µm (Agilent), mobile phases consisted of 5 mM ammonium formate in water (pH 2.3 with Trifluoroacetic Acid (TFA)) and 5 mM ammonium formate in acetonitrile (pH 2.3 with TFA), oven temperature was 50°C, injection volume 10 µL. Gradient elution was performed starting with 1 % solvent B and 1.4 mL/min held for 2 min, increased to 20 % B within 0.5 min, increased to 40% B within 3 min, further increased to 100% B within 2.5 min, held for 2 min at 2.2 mL/min. The initial conditions were then restored within 0.2 min and held for 1.8 min.

Dexamethasone, ketamine, rocuronium, adrenaline, noradrenaline and naloxone were diluted with 0.9 % NaCl-solution (injection concentration between 8 and 200 µg/mL); amiodarone was diluted 1:2000 (v:v) with methanole and for acetazolamide a solution of approx. 100 µg/mL in 0.9 % NaCl was prepared. Three dilution-samples were prepared from each experiment ampule and from one of the reference ampules of each substance. All measurements were recorded as the percentage of drug concentration compared to the reference drug concentration.

**Abbreviations**

**HPLC-DAD-MS/MS** – High-Performance Liquid Chromatography – Diode Array Detector – Tandem Mass Spectrometry

**TFA** – Trifluoroacetic Acid
